# Playing Piano Can Improve Upper Extremity Function after Stroke: Case Studies

**DOI:** 10.1155/2013/159105

**Published:** 2013-02-24

**Authors:** Myriam Villeneuve, Anouk Lamontagne

**Affiliations:** ^1^School of Physical and Occupational Therapy, McGill University, Montreal, QC, Canada H3A OG4; ^2^Feil and Oberfeld Research Centre, Jewish Rehabilitation Hospital, Research Site of the Montreal Center for Interdisciplinary Research in Rehabilitation (CRIR), 3205 Place Alton-Goldbloom, Laval, QC, Canada H7V 1R2

## Abstract

Music-supported therapy (MST) is an innovative approach that was shown to improve manual dexterity in acute stroke survivors. The feasibility of such intervention in chronic stroke survivors and its longer-term benefits, however, remain unknown. The objective of this pilot study was to estimate the short- and long-term effects of a 3-week piano training program on upper extremity function in persons with chronic stroke. A multiple pre-post sequential design was used, with measurements taken at baseline (week_0_, week_3_), prior to (week_6_) and after the intervention (week_9_), and at 3-week follow-up (week_12_). Three persons with stroke participated in the 3-week piano training program that combined structured piano lessons to home practice program. The songs, played on an electronic keyboard, involved all 5 digits of the affected hand and were displayed using a user-friendly MIDI program. After intervention, all the three participants showed improvements in their fine (nine hole peg test) and gross (box and block test) manual dexterity, as well as in the functional use of the upper extremity (Jebsen hand function test). Improvements were maintained at follow-up. These preliminary results support the feasibility of using an MST approach that combines structured lessons to home practice to improve upper extremity function in chronic stroke.

## 1. Introduction

Persistent contralateral motor impairments are common following a stroke. It is estimated that 80% to 95% of patients experience sensorimotor upper extremity impairments as well as activity and participation limitations, which persist beyond 6 months after stroke onset [1]. This is a major concern as in order to manage daily activities, chronic stroke survivors often use nonoptimal compensation strategies that can lead to a pattern of learned disuse of the paretic arm and further exacerbate the level of disability. Existing therapies that aim at improving upper extremity function show modest to moderate improvements [2], possibly due to insufficient training intensity [3] and lack of adherence. It was also shown that well beyond the optimal recovery window that occurs within the first 6 months after a stroke, rehabilitation still has the potential to induce neurological and functional changes [4, 5]. There is a need to develop and implement interventions that will meet the patient's interests to actively engage them during and beyond the supervised rehabilitation period so that long-term improvements in upper extremity function can be achieved.

Music-supported therapy (MST) is an innovative approach that has been shown to yield larger improvements in fine and gross motor dexterity compared to conventional rehabilitation and constraint-induced movement therapy in acute stroke survivors [6]. MST was also shown to yield enhanced motor skills and neuroplastic changes of auditory-motor network in chronic stroke participants [7]. In addition to integrating key principles of motor learning and providing instantaneous auditory feedback on performance, the rapid establishment of auditory-motor coupling during music playing would underlie the efficacy of MST [7, 8]. Such coupling can be observed within 20 minutes of musical training and is largely enhanced after 5 weeks of training in nonmusicians [9]. Existing MST programs, however, involve 5 days/week of training and may be difficult to implement in an outpatient and community rehabilitation settings. Furthermore, no previous MST program has focused on finger movement accuracy, timing, and speed, which are important determinants of finger coordination. We have developed, using a user-friendly computerized piano program, a piano training paradigm that provides feedback on note accuracy, timing and speed while allowing participants to progress through finger sequences of increasing complexity. The purpose of this study was to investigate the feasibility of an individually tailored piano training intervention that targeted finger movement coordination and combined structured piano lessons to home practice. The specific objective was to estimate the short-term and retention effects of a 3-week piano training program on manual dexterity, finger movement coordination, and functional use of upper extremity in persons with chronic stroke.

## 2. Methods

Three male participants with a mild to moderate deficits of upper extremity motor function due to a first supratentorial chronic stroke (6 to 24 months duration) in the middle cerebral artery territory were recruited after being discharged from rehabilitation (Table 1). Participants had (1) some capacity of dissociation of upper extremity movements as reflected by scores of 3 to 6 on the arm and hand components of the Chedoke-McMaster Stroke Assessment and (2) the ability to follow simple instructions. They had corrected to normal vision and were free of visual field defects (Goldman perimetry), hemineglect (<6 omissions, Bell's test), and cognitive deficits (scores > 23, Montreal Cognitive Assessment). None had musical experience. The study was approved by the Ethics Committee of the Centre for Interdisciplinary Research in Rehabilitation (CRIR), and informed consent was obtained from each participant. 

Subjects participated in a step-by-step musical training consisting of three individual 1-hour sessions per week for 3 consecutive weeks, for a total of 9 sessions. The individual sessions were complemented with a home program consisting of biweekly piano exercises of 30 min duration. Synthesia, an MIDI piano program, was used to program and display the musical pieces played by the participants on the electronic piano keyboard (Yamaha P155) during the training sessions. The musical pieces involved all 5 fingers of the paretic hand, and participants were cued to press the piano key(s) indicated by the visual stimuli (illuminated, blue dot) presented on the computer screen. Nine musical pieces were created and were introduced to the participants in an increasing order of difficulty: (1) *simple*, or “following notes” involving movements of consecutive fingers; (2) *intermediate,* or third, fourth, and fifth intervals involving movements of nonconsecutive fingers; and (3) *complex, *which involves chords, that is 2 fingers played at the same time. Within each musical piece, the participants started at a tempo of 30 bpm. When reaching a note accuracy and timing score of 80%, as measured in Synthesia, the tempo increased by steps of 10% until reaching a tempo of 60 bpm. Home piano exercises were executed on a roll up flexible piano (Hand Roll Piano 61 K).

Changes in fine motor (nine hole peg test (NHPT)) and gross motor dexterity (box and block test (BBT)) were measured at multiple baseline time points (week_0_, week_3_), immediately prior to (week_6_) and after the intervention (week_9_), and at a 3-week follow-up (week_12_). The Jebsen hand function Test (JHFT), which reflects the functional use of the hand, is time-consuming and was administered only at pre- and post-intervention, as well as follow-up. Piano performance measures, including timing and note accuracy, were collected with Synthesia throughout the training sessions. Participants recorded their home practice duration and frequency in a logbook.

## 3. Results

All participants showed improvements in note accuracy and timing accuracy within and across the training sessions. Participant 3 completed 3 musical pieces and the two others completed 5 pieces during the 3-week intervention. They progressed through finger sequences of increasing complexity, involving movements of consecutive fingers followed by movements of nonconsecutive fingers (intervals). Each musical piece started at 30 bpm and were practiced on an average of 25 times over 2 to 3 training sessions before reaching a note accuracy > 80% at 60 bpm. When considering a tempo of 60 bpm, the duration of musical pieces also increased from 17 s to 48 s (participants no. 1 and no. 2) and from 17 s to 32.5 s (participant no. 3) between the first and last training session.

**Figure 1:**
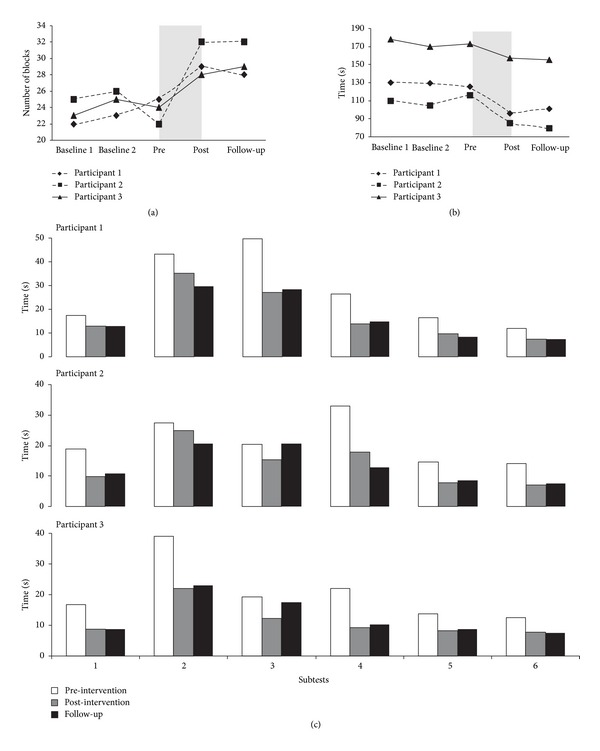
Scores of the three participants at different time points on the box and block test (a), nine hole peg test (b), and the Jebsen hand function test (c). For the box and block test, the scores represent the maximum number of blocks transported from one box to the other in 60 seconds. For the nine hole peg test, the time required to place and remove nine dowels into a nine holes is represented. For the Jebsen hand function test, the time required to complete the following subtests is shown: (1) simulating page turning; (2) lifting small common objects; (3) simulated feeding; (4) stacking checkers; (5) lifting large, light objects; (6) lifting large, heavy objects.

A mean increase of 6 blocks (range: 4–10 blocks) and a mean reduction of 24.8 s (16–31 s) were observed on the BBT and NHPT, respectively, between pre- and post-intervention (Figure 1). At variance, little variations were observed between baseline measurements at week_0_ (BBT = 23.3; NHPT = 139.2 s) and week_3_ (BBT = 24.7; NHPT = 134.8 s) and pre-intervention (BBT = 23.7; NHPT = 138.1 s). None of the participants were able to complete the writing subtest of the JHFT. The 3 participants, however, showed larger scores on all other subtests of the JHFT at post-intervention compared to pre-intervention, with mean increments ranging from 36.2% to 44.2% (Figure 1). Post-intervention scores for the BBT, NHPT, and JHFT were maintained at the 3-week follow-up. Average home practice duration was 50 minutes per session, which exceeded the 30 minutes practice time required. All participants reported enjoying the piano training program, and they all expressed the desire to continue piano lessons after their participation in the study. No adverse reactions to the intervention were reported, with the exception of one participant (no. 2) displaying occasional “hand stiffness” typical of spasticity during the intervention, as well as a fatigue described as a “general fatigue” after the training sessions. The stiffness was going away with frequent breaks, and the fatigue resolved within a few hours after the sessions.

## 4. Discussion

This case study is, to our knowledge, the first to report the immediate and retention effects of a structured piano training program combined to home practice in chronic stroke survivors. Improvements in fine and gross manual dexterity, as well as in the functional use of the hand, were observed in all three participants immediately after, but also at the 3-week follow-up. These changes were accompanied by improvements in the speed of execution, as well as in the timing accuracy and note accuracy for each musical piece. Such positive training effects are especially remarkable, considering that participants involved in this study were suffering from a chronic stroke and were exposed to an intervention of short duration. Similar improvements in manual dexterity were observed in a 3-week MST program involving piano and drum pad playing in acute stroke survivors [6], although changes in fine dexterity in the present study (NHPT: +24.8 s) do appear to exceed those reported in the combined drum-piano MST paradigm (NHPT: +13 s). Present results contrast, however, with findings from a case report involving the combined drum pad and piano playing intervention in chronic stroke survivors, where no changes on the NHPT and BBT were observed following the intervention [10]. We hypothesize that the impact on manual dexterity observed in the present study is attributed to the intensity and specificity of the piano exercises that were specifically designed to target dissociated and coordinated finger movements, with an emphasis on note and timing accuracy, as well as speed of execution. Rich feedback was provided to the participants throughout the supervised training session using a computerized program that provided knowledge of performance (note accuracy and timing) and knowledge of result (final speed and error score).

Comparison of present training effects with other existing upper extremity interventions in chronic stroke such as constraint-induced movement therapy is difficult, due to the use of different outcome measures and inclusion of participants with different characteristics. Present findings, however, can be interpreted in the light of the smallest real differences, or true changes, for the BBT (6 blocks) and the NHPT (32.8 s) [11]. Despite of their chronic stage, our participants either approached (nos. 1 and 3) or exceeded (no. 2) the smallest real difference on the BBT. Similarly, one participant (no. 1) almost reached the smallest real difference for the NHPT while the two others made more modest gains. These observations suggest that MST has the potential to yield real improvements in upper extremity function in chronic stroke participants with different levels of hand and arm motor recovery. The presence of an enhanced functional use of the upper extremity post-intervention also suggests that a better coordination of finger movements can impact on the upper extremity as a whole, and may be a prime target for rehabilitation. Finally, the persistence of positive effects at follow-up further indicates that improvements can be maintained even after the cessation of the training. Compared to other therapies such as constraint-induced movement therapy MST may be less time consuming and labor intensive. It has the potential to be safely self-managed and pursued well beyond the rehabilitation period, such that gains can be maintained or further enhanced.

MST relies on key principles of motor learning, including repeated and task-specific practice as well as the involvement of multisensory feedback, which gives instantaneous knowledge of result and performance. It would further take advantage of a rapid establishment of auditorimotor coactivation induced by the musical training [8], while engaging the participants in an individually tailored and rewarding program. Participant's remarkable adherence to the home practice sessions, which led to practice times beyond expectations, indicates high levels of motivation. This motivation, as well as a perception of being engaged in an enjoyable, socially valued leisure activity, are factors that may help patients pursuing musical lessons beyond the usual rehabilitation time frame.

The small sample size in this pilot study limits the generalization of results. Present results, however, support the feasibility of MST in chronic stroke and provide useful information that can be used to generate further hypotheses and design larger intervention studies. Longer-term benefits of the training on upper extremity function and quality of life should also be investigated.

## 5. Conclusion

This study provides preliminary evidence indicating that a piano training program combined to home practice is feasible and can lead to meaningful improvements in manual dexterity, finger movement coordination, and functional use of upper extremity in chronic stroke survivors. For the first time, it was also demonstrated that MST training effects are maintained at a 3-week follow-up. This unique intervention, which targeted finger movement coordination, engaged the participants in an individually tailored and highly motivating program. It has the potential to be self-managed and pursued on the long term, outside the rehabilitation setting, and lead to further and sustainable improvements in upper extremity function.

## Figures and Tables

**Table 1:** Initial participant characteristics.

	Participant 1	Participant 2	Participant 3
Age (years)	60	67	58
Gender (male/female)	Male	Male	Male
Time since stroke (months)	9	10	16
Side of stroke (left/right)	Right	Left	Right
Type of stroke (ischemic/hemorrhage)	Ischemic	Ischemic	Hemorrhage
CMSA arm/hand score (max = 7)	3/3	3/3	4/5
Piano experience (years)	0	0	0
Handedness	Right	Right*	Left*

*Affected hand is the dominant hand; CMSA: Chedoke-McMaster Stroke Assessment.
